# Breaking binary in cardiovascular disease risk prediction

**DOI:** 10.1038/s44325-024-00041-7

**Published:** 2025-01-13

**Authors:** Yichi Zhang, Akl C. Fahed

**Affiliations:** 1 Department of Medicine, Massachusetts General Hospital, Boston, MA USA; 2 Cardiovascular Research Center, Massachusetts General Hospital, Boston, MA USA; 3 Cardiovascular Disease Initiative, Broad Institute of MIT and Harvard, Cambridge, MA USA

**Keywords:** Cardiology, Cardiovascular diseases, Cardiovascular genetics

## Abstract

Atherosclerotic cardiovascular disease (ASCVD) remains the leading cause of death in the world. However, advances in genetics, omics research, machine learning (ML), and precision medicine have inspired revolutionary new tools in ASCVD risk stratification. Together, polygenic risk scores (PRS) and composite ML-based algorithms help shift the paradigm away from binary predictions towards more comprehensive continuum models. Continued efforts are needed to address socioeconomic and racial disparities in the PRS space.

## Introduction

Atherosclerotic cardiovascular disease (ASCVD) remains the leading cause of mortality and morbidity with staggering burden on the economy^1^. For example in the United States, over $320 billion in healthcare costs can be attributed to ASCVD alone, which has risen by 50% in the past two decades and is projected to rise steadily by year as the average US population continues to grow older^2^. Similar trends are seen in the UK^3^, Canada^4^, China^5^, Japan and Korea^6^, across South America^7^ and Africa^8^, as well as the rest of the world.

Thus, a great deal of public health efforts have been invested towards prevention of ASCVD and the management of various risk factors such as hypertension, diabetes, and hyperlipidemia^9^. Clinical risk calculators such as the ASCVD Pooled Cohort Equations (PCE)^10^, QRISK2^11^, and more recently PREVENT^12^, are used to estimate risk over an interval of time using a variety of binary and continuous measures of risk factors as inputs. While the output of those risk calculators is a probability of ASCVD over a fixed interval or over the lifetime, clinical guidelines use binary cut-offs to convert them to actionable recommendations, such as initiation of statin with PCE 10-year ASCVD risk estimate that exceeds 7.5%^13^. To add more complexity, multiple other risk factors, which are often not part of the calculator, are then layered on top as “ASCVD risk enhancers”, adding to an already intricate process of clinical decision-making. Those include binary factors such as family history and preeclampsia and continuous factors such as coronary artery calcium (CAC) score and lipoprotein a [Lp(a)]^14^.

Since most ASCVD clinical risk factors are continuous measures of exposure with a well-defined relative risk curve^15,16^, why are we obsessed with binarizing risk stratification?

The emergence of polygenic risk scores (PRS) as helpful tools for further stratification of ASCVD risk beyond traditional clinical predictors is contributing towards a shift away from binary thinking and towards personalized management. ASCVD is a highly heritable trait and PRS quantifies this genetic susceptibility and is available earlier in the life course, prior to the onset of risk factors. By design, PRS is a continuous measure that is normally distributed in the population and stratifies people across a gradient of risk. However, as clinical utility of PRS is advancing, it has become common practice to also group individuals, sometimes arbitrarily, into percentile groups of risk to qualify high and low genetic risk, simulating the binarization of clinical risk factors^17–19^. Is binarization of risk the only way for interpretability and implementation? Genetic susceptibility might provide an opportunity to revisit our binary approach to risk stratification.

**Fig. 1:**
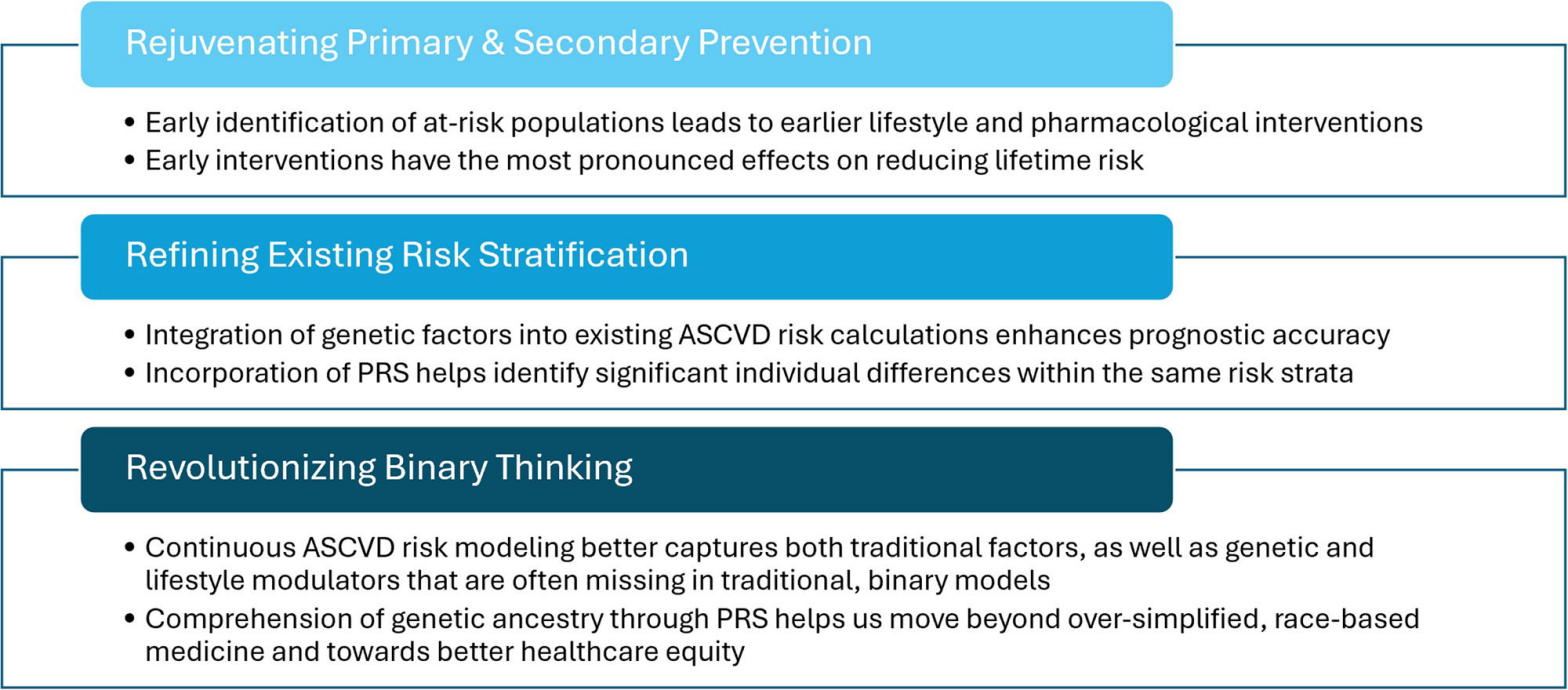
Clinical utility of PRS applications in ASCVD. Summary of key advantages offered by applying PRS in ASCVD risk models.

In this narrative review, we explore how large-scale data informing the continuum of risk for both clinical and genetic factors provide an opportunity to break binary and re-imagine precision in risk estimation (Fig. 1).

## Methods

Literature search and review were conducted via PubMed and EBSCO libraries. Search terms included individual and combination terms from the following topic list: “polygenic risk score/PRS”, “Genome-wide Association Studies/GWAS”, “atherosclerotic cardiovascular disease/ASCVD”, “risk stratification”, “risk modeling”, “pooled cohort equations/PCE”, “binary”, “continuous”, “machine learning”.

Only articles originally written in English and published in a peer-reviewed journal were included in this review. Due to the novel nature of the topics reviewed, some ongoing studies were also cited, and we acknowledge that final data analysis and study results are still pending, as the evidence base around cardiovascular risk prediction continues to evolve.

### Development and validation of PRS in ASCVD

The initial wave of genetic loci identified and linked with ASCVD were mostly monogenic risk variants^20^. These discrete loci consisted of important coding sequences that when disrupted, conferred a significant risk for atherosclerotic disease through different mechanisms. Some of the most well-known genes containing such variants include the aforementioned Lp(a) and ApoB^21^, as well as proprotein convertase subtilisin/kexin type 9 (PCSK9), which has already been successfully used as a pharmaceutical target^22^. While these monogenic variants can certainly lead to severe hypercholesterolemia and predispose individuals for premature atherosclerosis, their prevalence at the population level is overall low, with few loci exceeding >1% prevalence^23^. And since these monogenic variants tend to follow Mendelian inheritance patterns, their presence can be suspected through typical family history screening questions. However, monogenic variant-related disease only accounts for a small fraction of patients who develop ASCVD: most patients’ risk is not attributable to a single gene mutation, and most also do not have a clear familial history pattern to refer to. Thus, significant efforts were made to explore the genetic risk puzzle beyond these monogenic variants.

The quest for the development of PRS became possible with the evolution in large biobanks, genotyping technology, and computing power, allowing for genome-wide association studies (GWAS)^24^. PRS synthesize risk calculation based on weighted summation of previously discovered variants associated with ASCVD^25^. The underlying reasoning is simple yet elegant: even when individuals do not have a single focal mutation of large effect, a combination of smaller effect variants from numerous loci can add up to clinically significant ASCVD risk^18,19,26^. This phenomenon would also help explain severe ASCVD seen in patients with no apparent familial risk and previously negative monogenic screening^27–29^.

One of the first steps to synthesizing a PRS is to interpret and prune through effect size data of millions of variants from GWAS^30^. For example, certain variants might be excluded based on lower statistical significance (in the case of *p-value* thresholding), propensity for linkage disequilibrium (since certain variant pairs are geographically closer to one another within a chromosome), or sometimes both^17,31^. Overall, there is considerable variation in the statistical methods used for PRS calculation (including but not limited to PRS-CS, LD-pred, metaGRS, Lassom regression etc.) and no consensus about an optimal algorithm has been reached^17,32^. After PRS derivation is complete, validation is typically carried out in a separate patient population to ensure generalizability^17,33^. Often, multiple PRS are calculated for each individual within the study cohort and a best fit is chosen based on their respective predictiveness^17^.

In light of this tremendous progress in genomic medicine and its growing applications, it becomes especially worthy to note that traditionally GWAS and subsequent PRS derivation and validation have been conducted in patient populations with predominant European ancestry, as well as those of higher socioeconomic status and greater access to healthcare^34^. Specifically, there has been disproportionately low representation of patients of African ancestry, those from lower socioeconomic strata and rural geographical areas in genetic studies^35^. This might lead to less sophisticated genetic understanding of these traditionally underserved patients. Today, increasing representation in datasets and developing methods that improve cross-ancestry performance in PRS is a priority in genomics research.

### Clinical utility of PRS in ASCVD

GWAS and subsequent PRS derivation and calculation have helped identify more and more patients at risk for CAD, especially younger patients with subclinical CAD who were previously considered to have no traditional risk factors or modifiable comorbidities^36,37^. For example, when PRS for CAD was calculated for individuals of the CARDIA study, which consisted of young adults aged 18–35 who were followed longitudinally for over two decades. Wells et al. found that PRS for CAD was able to help predict increased coronary artery calcium score (CAC), especially when used in conjunction with emerging modifiable risk factors^36^.

These results can have significant implications for primary prevention. Young patients without obvious risk factors but who have a high PRS for CAD may be candidates for more proactive lifestyle modification counseling, early initiation of lipid-lowering therapy, as well as imaging to identify subclinical CAD^38,39^. In an analysis by Agaram et al. if a high PRS had been formally included as an indication for initiation of lipid-lowering therapy for primary CAD prevention, an extra 4.1% of patients would qualify for statins^20^. In Australia and New Zealand, the planned ESCALATE study will be a prospective trial using PRS to identify high-risk individuals for CAC scan during regular primary care visits and should shed light on the efficacy and feasibility of real-world PRS-CAD screening^40^. Our group is also leading the PROACT clinical trials (NCT05819814, NCT05850091), which are exploring detection of subclinical coronary atherosclerosis using coronary CT angiography among individuals with high PRS, and subsequent targeting with behavioral and pharmacological interventions to improve cardiovascular health and mitigate plaque progression^41^.

In the past decade, there have been growing applications of PRS in primary prevention of CAD and many other common comorbid diseases, interest has keenly grown around personalized risk stratification as a complement to conventional risk factors. Both Khera and Inouye et al. highlight this versatility in their landmark papers published in 2018 with data from the UK BioBank^19,42^. Since, there has been a cornucopia of studies that have built-on and improved upon the performance of existing PRS models, and especially their performance across multiple non-European ancestries. For example, Patel et al. demonstrated a 1.73 hazard ratio (HR) per standard-deviation increase in PRS in a study population that had good representation of African, Hispanic, and South Asian ancestry in addition to the majority European population^18^. We have also indicated improved performance of multiple cardiometabolic PRSs in Middle Eastern populations and described pragmatic methods to leverage existing data to optimize for diverse population groups^17,43^. Specifically, there has been a focus on improving risk stratification within individuals of the same PCE risk cohort, which can potentially lead to more individualized management decisions and better outcomes. For example, there is a significant gradient of CAD risk within the same PCE ASCVD risk stratum, and the addition of the PRS improves the C-statistic of the PCE model^18,29^. Consistently, integrated risk models that combine PRS and clinical risk such as PCE result in the best performance, especially in individuals under the age of 55 years^39,44^. To summarize, PRS not only helps with early identification of high-risk segments of the population but also with improved and personalized stratification of patient cohorts that are traditionally considered to have similar ASCVD risk, as well as maximally reducing event risk in those with established ASCVD by individualizing lifestyle and medication interventions^45^.

For patients with existing intermediate-high risk for CAD compounded by high PRS, it may be reasonable to target lower LDL-C levels by up-titrating statins, adding bile acid sequestrants, or PCSK-9 inhibitors to achieve more aggressive lipid control^46^. PRS may also serve as an additional piece of clinical data to help inform pre-test probability and guide the triage process for deciding between different modalities of cardiac stress testing.

Another key utility of PRS in CAD lies with its ability to guide personalized pharmacotherapy. PRS can help identify groups of patients who are more likely to benefit from specific treatments or interventions than the general population. For example, Natarajan et al. used a PRS-CAD score derived from 57 variants and identified a group of high-risk patients that benefit significantly more from statin therapy than all others (46% vs. 26% relative risk reduction in non-fatal myocardial infarction or death resulting from CAD)^47^. Damask et al. report that treating patients with a history of acute coronary syndrome (ACS) and a high PRS-CAD with alirocumab, a PCSK-9 inhibitor, resulted in much greater reduction in major adverse cardiac cardiovascular events (MACE) [HR = 0.63; *p* = 0.004] as compared to its effect in those with lower PRS-CAD (HR = 0.87; *p* = 0.022)^48^. Marston et al. also that patients with high genetic risk for CAD derived the greatest benefit from PCSK-9 inhibitor therapy regardless of the presence of clinical risk factors^49^. In addition, the pharmacotherapy, Khera et al. also found that among those with high genetic risk for CAD, a favorable lifestyle was associated with a 46% relative risk reduction in coronary events when compared to an unfavorable lifestyle^50^. Furthermore, Maamari et al. show that providing genetic risk assessment information in preventative clinics resulted in a change in management for 40% of patients, including medication changes and pursuing further imaging^27^. Because of the incredible potential of PRS applications in preventative medicine settings, there have been large-scale, ongoing efforts to better characterize, report, and implement PRS across patients from diverse ethnic backgrounds. These include the All of Us initiative, which has launched in 2018 with over 340 recruitment sites and a goal enrollment of 1 million participants^51^, as well as the eMERGE (Electronic Medical Records and Genomics) network study^52^, which has started enrollment since 2022. A summary of the clinical utility of PRS in ASCVD can be found in Fig. 1.

### Breaking binary for clinical risk factors

Over the past few decades, while there have been increasing research efforts rallied around clinical risk stratification, most models that clinicians routinely use (PCE, HEART, TIMI, CHA_2_DS_2_-VASc, etc.) only account for a limited selection risk factors/variables^53^. The advantage to these existing models lies with their convenience and ease of use, but the true accuracy of these risk stratifications is often not ideal^54^.

The first major flaw of current cardiovascular risk calculators is their binary stratification of input variables. A patient with diabetes and an HbA1c of 15% is often accounted for the same way as another patient with an HbA1c of 7%^55^. However, most risk factors for ASCVD, such as HbA1c, blood pressure, LDL-C, BMI, exist on a vast continuum rather than the overly simplified, binary variables that they are currently represented by. Additionally, the liability thresholds that are used for each clinical risk factor do not necessarily represent a true biological or pathological cutoff, but rather populational-level data integrated with expert consensus for ease of use and stratification^56^. Does a “non-diabetic” patient with an HbA1c of 6.3% truly have significantly lower risk of ASCVD than a patient with diabetes and an HbA1c of 6.4% as current risk calculators may suggest? Clinicians can synthesize information holistically and determine that these patients’ disease risk is rather comparable in this scenario, and thus it is time for the risk calculators to reflect this continuum of risk more accurately. This can drastically improve stratification and change clinical decision-making for patients, especially those who fall close to the traditional thresholds and are thus at a high risk of being misclassified.

For example, studies have shown that HbA1c is positively associated with CAD risk as an independent, continual variable^57^ and that the incorporation of HbA1c in addition to diabetes as a binary variable greatly enhances CAD prediction^58^. LDL-C levels at or below the current threshold of hyperlipidemia have also been shown to independently correlate with atherosclerosis^59^, which again demonstrates flaws of a binary LDL-C threshold. The popularization of cholesterol particle measurements such as ApoB^60^ and Lp(a)^61^ also calls for revolutionizing upon the traditionally LDL-C-centric perspective. Similarly, there have been efforts to halt the dichotomization of BMI among other continuous variables to better qualify their true effect sizes^62^. To this end, the American Heart Association has also highlighted the incremental predictability of PRS in atrial fibrillation, CAD, hypercholesterolemia, type 2 diabetes, venous thromboembolism among other diseases in their 2022 scientific statement^63^.

Historically, binary stratification of clinical risk variables was widely accepted and implemented due to its ease-of-use^64^. Before the onset of the digital era of medicine, clinicians relied on paper-based medical records and had extremely limited access to any computational platform. Thus, the computation of clinical risk relied mostly on each individual clinician’s anecdotal experience. The introduction of simple binary clinical risk calculators (e.g., from the Framingham Study in 1976 or the CENTOR criteria in 1981)^65,66^ served to standardize clinical decision-making during a time where subjectivity bias was commonplace. They included only a few risk factors (age, sex, smoking status, hypercholesterolemia, hypertension status) and were easy to tabulate even without a calculator or computer and thus there was a relatively low barrier for uptake among clinicians. In this way, binary risk stratification models contributed significantly to the widespread implementation of risk calculators and helped to standardize cardiovascular care, especially in the preventive cardiology space. However, as electronic medical records became mainstream, modern risk calculations can be conducted in a more sophisticated manner, leading to more accurate predictions than their binary predecessors. In one recent example, machine learning of the electronic medical records was used to generate an in-silico marker for CAD and risk of death on a continuous spectrum^67^. However, the force of clinical inertia is strong and changing the lifelong work habits of clinicians will take significant time and effort^68^. Thus, it becomes even more imperative to revamp the curriculum on clinical risk stratification at every stage of medical training to better reflect the latest research and to lay down a solid foundation for the next era of personalized medicine.

### Breaking binary through polygenic risk scores

Genetic susceptibility to disease is present from time of birth, and accounting for that susceptibility might help quantify lifetime exposure rather than single biomarker measurement. Incorporation of genetic variables to properly account for lifetime exposure might help revolutionize current binary risk stratification. Traditional input variables such as blood pressure, HbA1c, LDL-C and BMI are typically measured at clinic visits and can represent only a snapshot in time of a patient’s overall health profile and fail to account for longitudinal exposure. Despite this flaw, these inputs are often used in equations that seek to compute 10-year or even lifetime risk, which can introduce bias and inaccuracies. The addition of genetic variables might better account for lifetime exposure of each of those risk factors.

For example, Cho et al. showed that blood pressure PRS was able to improve identification of patients at higher risk of MI, stroke, and other major cardiovascular events regardless if they had normal BP, untreated or treated hypertension^69^. In fact, the PRS was able to identify 10% of those with normal BP had similar cardiovascular risk to those with untreated hypertension- a crucial risk that traditional, binary risk calculators would likely fail to capture, thereby missing opportunities for additional intervention. Khera et al. demonstrated similar findings in LDL-C, where variants in familial hypercholesterolemia (FH) in genes in patients of all LDL-C levels, normal or elevated, resulted in higher risk of coronary artery disease^70^, and these findings were later echoed by Zhang et al.^71^. This suggests the significant utility of genetic screening in addition to LDL-C-based risk stratification to better compute lifetime cardiovascular risk. PRS has also been successfully used to further sub-stratify HbA1c-predicted risk for CAD, stroke, and atrial fibrillation^72^. Patients who had a top-tertile PRS were found to have considerably higher risk for CAD despite a normal HbA1c (<6.4%) when compared to patients with diabetes but with lower-tertile PRS, a prediction that traditional risk calculators would be unable to perform. Additionally, PRS has also been used to predict both the incidence of renal failure^73^ as well as rate of decline of glomerular filtration rate in patients with chronic kidney disease^74^. In coronary artery disease, there is emerging data supporting the notion that PRS is associated with faster plaque progression and increased risk of recurrent events even among those with known disease^26,75^. There have also been investigations into PRS’ utility in predicting BMI and risk stratification for obesity, as well as how metabolic syndromes interact with active or inactive lifestyles differentially based on genetic predisposition^76,77^. We recently showed that genetic risk and obesogenic lifestyle jointly impact the risk of obesity and its comorbidities^72^. To this end, there have already been real-world feasibility and implementation studies for continuous models integrated with PRS in both the cardiovascular^44,78^ and oncologic^79^ realms that describe their usefulness in incrementally improving the accuracy for disease risk prediction.

For example, a real-world feasibility study on integrating PRS and continuous modeling with existing risk calculators for ASCVD in a primary care setting was performed by Fuat et al.^78^ In a cohort of 800+ patients across the UK, they found that both an overwhelming percentage (>90%) of physicians and patient participants found the integrated risk estimation tool straightforward and helpful. The integrated tool also helped change management for over 25% of patient participants, especially when they were estimated to have higher risk with the new model. The fact that this integrated model was successfully implemented in a primary care setting is also significant as this is the most frequent access point that the healthcare system has with most patients. Another real-world UK study found an overall net reclassification improvement of 5.9% when using an integrated model for CAD prediction, and up to an impressive 15.4% for middle-aged male patients^44^.

In addition to the incorporation of continuous risk factors, future prediction systems can further improve by integrating data from different time points in patients’ lives, contributing to a more dynamic and longitudinal model. For example, Yang et al. incorporated 20 years’ worth of blood pressure data into the CV risk prediction model for a primary care cohort^80^. More recently, Yu et al. used 8 years of longitudinal risk factor data in an integrated, machine-learning model, and improved upon the predictability of the PCE by a net reclassification index of 0.385^81^. The incorporation of such longitudinal datapoints is made possible by the popularization of electronic health records and can offer incremental utility in future ASCVD prediction models.

**Fig. 2:**
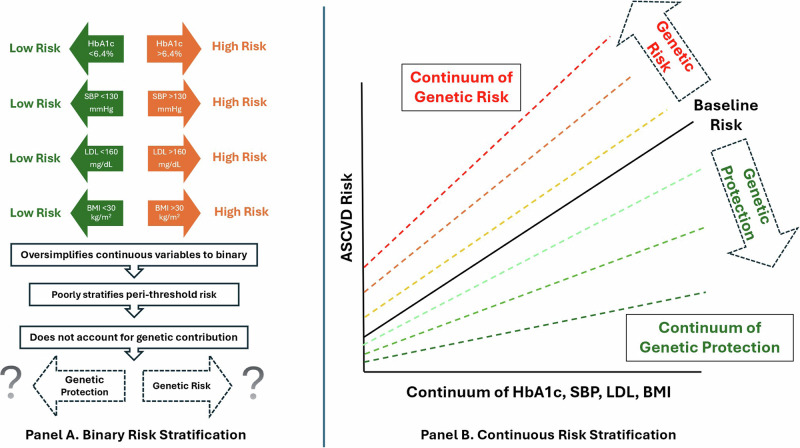
Breaking binary in cardiovascular risk stratification. **A** Demonstrates a traditional, binary cardiovascular risk stratification model. **B** Demonstrates a novel, personalized, continuous stratification model more to accurately reflect cardiovascular risk, effectively circumventing traditional, arbitrary threshold values. It also accounts for genetic factors that significantly modify upon acquired or lifestyle risk factors.

**Fig. 3:**
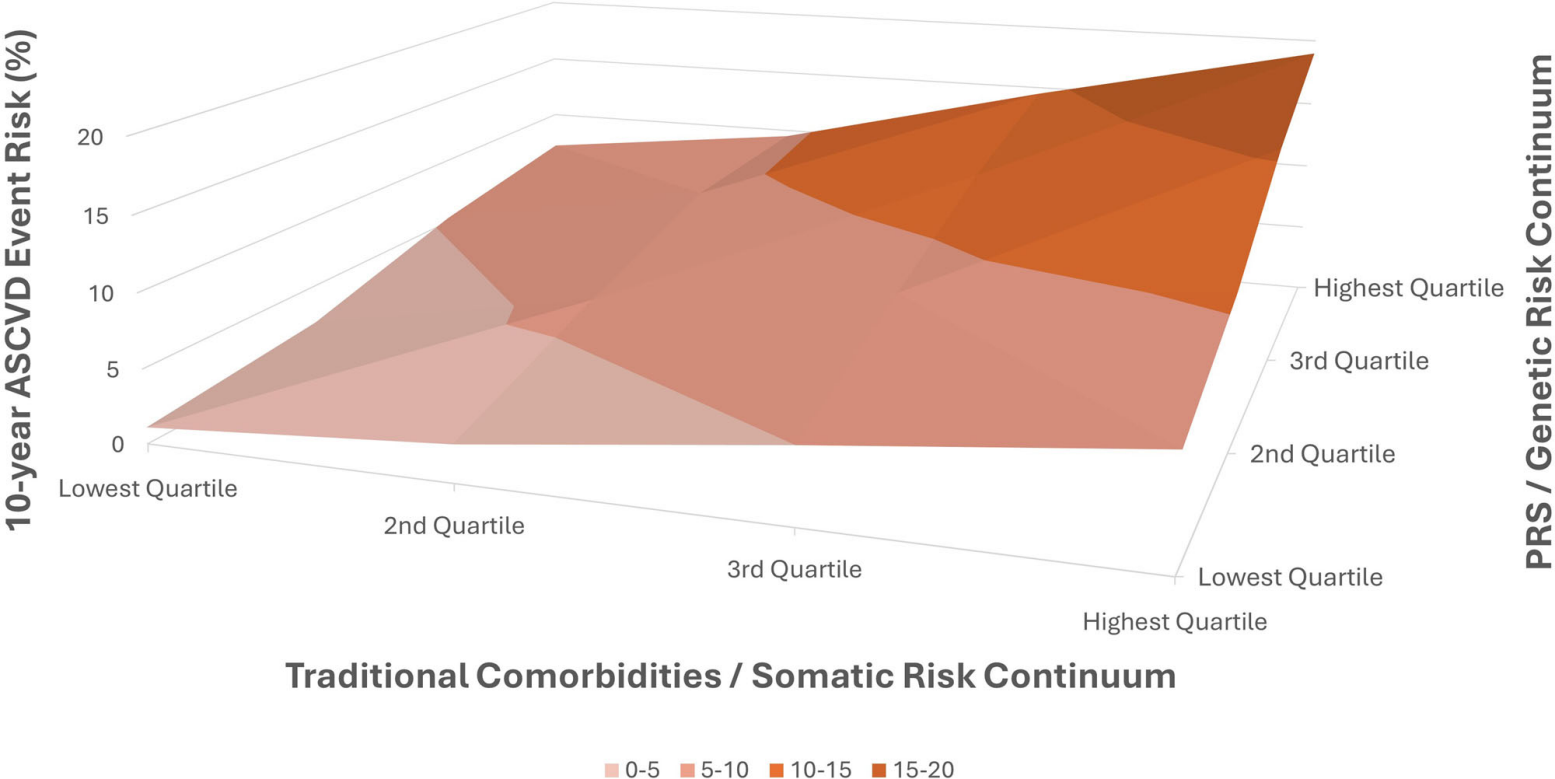
Graphical representation of the integrated ASCVD risk prediction model. Integrating PRS and genetic factors with traditional comorbidities on a dual continuum leads to more accurate and personalized ASCVD risk prediction.

By design, PRS is a continuous risk factor, and by studying how to best integrate genetic factors and PRS into existing cardiovascular risk prediction models, we can envision a future that shifts away from the traditional, binary paradigm and towards a more comprehensive and personalized outlook for preventive cardiology (Fig. 2 and Fig. 3).

As a relatively novel and evolving tool in the ASCVD prediction space, PRS may encounter several barriers to widespread implementation^82^. First, there is currently limited accessibility to genetic testing nationwide. Genetic testing is seldom available outside of academic institutions in larger metropolitan areas, especially newer tests that may require specialized equipment for analysis. Among current genomic studies, there is also a significant under-representation of patients with non-European ancestries, resulting in limited generalizability in more racially and ethnically diverse populations^83^. Future genomic investigations should thus place emphasis on inclusivity and diversity of patient populations studied to minimize systemic bias and improve generalizability of any clinically applicable results. Second, the low affordability and lack of insurance coverage for newer genetic tests may discourage patients from undergoing appropriate screening. Out-of-pocket costs for a single test may often range up to several thousand dollars, which would not be financially feasible for most patients. Third, there is currently suboptimal education of physicians on appropriate ordering and interpretation of genetic screening. Primary care doctors often have the most frequent touchpoints with patients but may not feel empowered to order or interpret genetic screening due to lack of training in this area. On the other hand, genomic medicine specialists and cardiologists with genomic training can be difficult for patients to routinely access. Such is why improving genetic screening education and training in the primary care setting would be imperative for its widespread implementation^84^. There are patient concerns about discrimination from disability or life insurance institutions. The Genetic Information Nondiscrimination Act (GINA) does not apply to these insurance subtypes, so patients may be wary about getting appropriate genetic testing as it could significantly affect their insurance candidacy and premiums. Finally, there is also risk for misunderstanding for both physicians and patients on the interpretation of PRS results. There is an inherent level of uncertainty associated with any type of risk estimation, and especially more considerations with the involvement of genetic information, which needs to be acknowledged and understood by both parties through effective education and counseling^85^.

### Breaking binary for the advancement of equitable, personalized medicine

Historically, race and ethnicity have always been used as divisive labels in medicine and different race-based thresholds have been established for various clinical risk factors. However, modern research shows that while these race-based thresholds may have been designed with the intention to personalize risk stratification, they may actually introduce controversial biases^86^. Most notably, new glomerular filtration rate equations that incorporate both creatinine and cystatin C but omitted race led to more accurate classifications, especially for African American patients, whose chronic kidney disease risks were disproportionately under-detected in the traditional risk-based equation^87^. On the other hand, a more positive result can be seen with ethnic-based BMI thresholds, where the use of a lower threshold to define obesity in Asian, South Asian, and Black patients helped identify additional patients at risk for developing diabetes^88,89^.

As genomic medicine undergoes rapid development and expansion, it becomes more crucial to recognize the widening of socioeconomic, racial, and geographic disparities in healthcare access and quality^90^. And like many novel medical technologies, indices, and devices that came before, genomic medicine accessibility is significantly affected by various socioeconomic drivers that have differentially affected patients of different races, ethnicities, and backgrounds. Genetic testing is disproportionately less available to patients from lower socioeconomic strata, racial minority groups, as well as those from rural areas or other regions with less convenient access to healthcare^91–93^. Additionally, for many minority patients, decades of mistrust, misinformation, and mistreatment by a systemically prejudiced national healthcare system have resulted in deep-rooted stigma regarding clinical trials as well as genetic tests^94^. These stigmas not only result in lower utilization of potentially life-saving genetic screening measures in this underserved population but also lead to less overall genetic understanding of minorities and further perpetuate this bleak cycle. Some of these trends are already being addressed with grassroots efforts for increasing diversity in genomic research, such as the All of Us Research Program, which currently includes 77% of its participants from under-represented communities in biomedical research and 46% of its participants from under-represented racial and ethnic groups^95^. These combined efforts all contribute towards the eventual completion of a more comprehensive and equitable genomic atlas for patients of all races and ethnicities.

With the incorporation of continuous variables into risk calculators, the use of controversial race- or ethnic-based thresholds could be circumvented altogether in favor of truly individualized risk estimation. For example, dimensionality reduction approaches can help better define principal components of genetic ancestry^96^. While conventionally, those principal components have been used to define clusters of known continental and subcontinental population groups^97,98^, a landmark paper by Novembre et al. emphasized the use of continuous principal component analysis (PCA) helps to better characterize the spatial variations within continental and subcontinental groups, and that improved interpretation of PCA can help correct for certain artifacts seen in populational models of the past^99^. More recently, there has been growing number of PCA toolkits developed for more efficient genomics research^100^. As we move away from the notion of race in medicine^101^, using continuous measures derived from large, distinct populations and communities that meaningfully capture an exposure of interest such as geography, genetic diversity, and socioeconomic status will mark an important step towards achieving a true synthesis between healthcare equity and personalized medicine.

Thus, it is more important than ever to advocate for the ethical and equitable expansion of genomic medicine, including PRS applications, to all traditionally underprivileged and underserved patient populations. This advocacy is especially needed within the primary care setting. While PRS may be available in tertiary centers or specialist offices, primary care clinics are where the most frequent touchpoints with patients occur, and can thus potentially deliver the greatest populational impact^102^. In particular, studies have shown that predominantly minority-serving primary care physicians are less likely to have ever ordered genetic screening for their patients^103^. This could be the result of equal parts patient stigma and mistrust of genetic screening, as well as a lack of proper education and training of the clinician, and both problems warrant tremendous efforts to reconcile.

For example, the Genomic Medicine at Veterans Affairs (GenoVA) study is a randomized clinical trial launched in 2023 that seeks to investigate whether PRS expansion and implementation in a real-world primary care setting could help reduce time-to-diagnosis, alter management, reduce health disparities, and ultimately help improve clinical outcomes^104^. Positive trial results here can certainly help secure further public health infrastructure investment surrounding genetic screening, facilitate appropriate clinician awareness and training, and bolster future PRS implementation in primary care, especially within traditionally low-resource settings and underserved populations^105^.

## Conclusion

Over the past decades, traditional, binary clinical risk stratification served its purpose by helping to standardize care in an era where computing power was limited. However, modern clinicians, equipped with the appropriate education and technology, should take strides to revolutionize upon binary models and instead implement continuous risk prediction into their everyday practice to minimize biases, achieve better accuracy, and personalize management for every patient. These novel, continuous risk stratification models will also integrate genetic and lifestyle risk factors that were previously unaccounted for under the binary system.

To this end, genomic medicine and PRS provide exciting new frontiers for ASCVD risk stratification, prevention, and management. Specifically, the integration of PRS with traditional clinical risk factors can promote early prevention and screening measures in at risk populations, improve personalized risk assessment and prognosis within an existing clinical risk stratum, as well as help lower recurrent event rates in those with established disease by making recommendations regarding tailored medical or lifestyle interventions. It will also help us move beyond often oversimplified, race-based clinical practices, gain more understanding into the underlying genetics, socioeconomic, and geographical factors at play, and ultimately promote healthcare equity.

Future personalized risk models should seek to integrate PRS, traditional clinical risk factors, as well as surrogates of lifestyle elements such as data from digital health wearables regarding sleep, physical activity, and dietary habits etc.^106^. After all, the time that a physician spends with each patient in clinic is but a fraction of a glimpse into their overall health profile. And by capturing lifestyle factors accurately, models will be able to provide a much more comprehensive risk assessment and hopefully lead to important changes in screening or management decisions.

While clinical inertia poses a tremendous challenge in changing clinician habits and the widespread implementation of integrated, continuous risk prediction models will likely take momentous efforts, it all starts from awareness and education at every level of medical training. With renewed advocacy and the continual evolution of novel evidence, we believe that PRS and continuous risk modeling will play a decisive role in the promising era of personalized and precision medicine to come.

## Data Availability

No datasets were generated or analysed during the current study.
